# Movements of Wild Ruddy Shelducks in the Central Asian Flyway and Their Spatial Relationship to Outbreaks of Highly Pathogenic Avian Influenza H5N1

**DOI:** 10.3390/v5092129

**Published:** 2013-09-09

**Authors:** John Y. Takekawa, Diann J. Prosser, Bridget M. Collins, David C. Douglas, William M. Perry, Baoping Yan, Luo Ze, Yuansheng Hou, Fumin Lei, Tianxian Li, Yongdong Li, Scott H. Newman

**Affiliations:** 1 San Francisco Bay Estuary Field Station, Western Ecological Research Center, U.S. Geological Survey, 505 Azuar Drive, Vallejo, CA 94592, USA; 2 Patuxent Wildlife Research Center, U.S. Geological Survey, Beltsville, MD 20705, USA; E-Mails: dprosser@usgs.gov (D.J.P.); bmcollin6@gmail.com (B.M.C.); 3 Alaska Science Center, U.S. Geological Survey, Juneau, AK 99801, USA; E-Mail: ddouglas@usgs.gov; 4 Dixon Field Station, Western Ecological Research Center, U.S. Geological Survey, 800 Business Park Drive, Suite D, Dixon, CA 95620, USA; E-Mail: wmperry@usgs.gov; 5 Computer Network Information Center (CNIC), Chinese Academy of Sciences, Beijing 100080, China; E-Mails: ybp@cnic.cn (B.Y.); luoze@cnic.cn (L.Z.); 6 Qinghai State Forestry Administration, Qinghai Lake National Nature Reserve (QLNNR), Xining 25700, Qinghai, China; E-Mail: houyuanseng@163.com; 7 Institute of Zoology (IOZ), Chinese Academy of Sciences, Beijing 100101, China; E-Mail: leifm@ioz.ac.cn; 8 Institute of Virology (WIV), Chinese Academy of Sciences, Wuhan 430071, China; E-Mails: litx@wh.iov.cn (T.L.); litx@wh.iov.cn (Y.L.); 9 EMPRES Wildlife Health and Ecology Unit, Animal Health Service, Animal Production and Health Division, Food and Agriculture Organization of the United Nations, Rome 00153, Italy; E-Mail: scott.newman@fao.org

**Keywords:** Brahminy duck, movement ecology, Tadorna ferruginea, avian influenza

## Abstract

Highly pathogenic avian influenza H5N1 remains a serious concern for both poultry and human health. Wild waterfowl are considered to be the reservoir for low pathogenic avian influenza viruses; however, relatively little is known about their movement ecology in regions where HPAI H5N1 outbreaks regularly occur. We studied movements of the ruddy shelduck (*Tadorna ferruginea*), a wild migratory waterfowl species that was infected in the 2005 Qinghai Lake outbreak. We defined their migration with Brownian Bridge utilization distribution models and their breeding and wintering grounds with fixed kernel home ranges. We correlated their movements with HPAI H5N1 outbreaks, poultry density, land cover, and latitude in the Central Asian Flyway. Our Akaike Information Criterion analysis indicated that outbreaks were correlated with land cover, latitude, and poultry density. Although shelduck movements were included in the top two models, they were not a top parameter selected in AIC*c* stepwise regression results. However, timing of outbreaks suggested that outbreaks in the flyway began during the winter in poultry with spillover to wild birds during the spring migration. Thus, studies of the movement ecology of wild birds in areas with persistent HPAI H5N1 outbreaks may contribute to understanding their role in transmission of this disease.

## 1. Introduction

The first outbreak of highly pathogenic avian influenza (HPAI) H5N1 in wild birds killed more than 6,500 birds in May 2005 at Qinghai Lake, China in the Central Asian Flyway (CAF; [1,2]). Five species died in the event, but the largest number of mortalities included bar-headed geese (*Anser indicus*) and ruddy shelduck (*Tadorna ferruginea*). The Anatidae are considered to be the reservoir for low pathogenic avian influenza strains ([3]; see summary in Takekawa *et al.* [4]). Following the outbreak at Qinghai Lake in 2005, HPAI H5N1 spread to Europe and Africa, and migratory wild birds, especially ducks, were implicated for this spread [1,2,4,5,6,7,8]. In subsequent years (2007 and 2009), similar HPAI virus strains were detected in wild birds in the spring and summer at Qinghai Lake [9], and repeated outbreaks were detected in the CAF [4,8].

HPAI H5N1 remains a major health issue to poultry worldwide with occasional cases of human mortality. China is one of the major poultry producing countries in the world, accounting for almost 20% of the global poultry production [10]. There have been major outbreaks of the disease in the poultry industry in over 20 provinces of China, which resulted in the death of over 30 million poultry during 2004–2008 either by infection or culling [11]. The country is also home to a large population of waterfowl, which are raised in open fields near lakes and rivers. This brings the domestic birds in close contact with the wild migratory birds that use these wetlands as breeding, staging and wintering sites. Few samples of HPAI H5N1 have been detected in thousands of living wild waterfowl, but epizootics in wild birds have continued to be recorded [8].

The ruddy shelduck (hereinafter shelduck) is an important migratory species in the CAF and was involved in the large outbreaks at Qinghai Lake where the virus was first detected. Over 140 shelducks died during the first reported outbreak of HPAI H5N1 at Qinghai Lake in 2005 [1,12]. It breeds at high altitude wetlands in the mountains of central Asia, and winters around lakes, rivers and other water bodies of south Asia [13,14]. It is also a winter migrant to Pakistan, Nepal, Bhutan, Bangladesh and Sri Lanka [15]. Globally, shelducks are distributed across Asia, Europe and Africa [16].

Shelducks migrate large distances between their breeding and wintering areas in Asia, and thus are potential vectors of disease. They breed in pairs or small groups around high altitude lakes in China, Mongolia and Russia where poultry densities are low or absent; however, they congregate in large flocks on their wintering grounds in the Indian sub-continent, southern Europe and North Africa, although these are considered separate populations without known connectivity [17]. Many of the lakes and other water bodies where they winter are in close proximity to poultry farms. Thus, susceptible individuals may pick up the virus and spread among other individuals, both domestic and wild, congregated in the wintering areas. Since shelducks were one of the major species of waterfowl that died during the 2005 Qinghai Lake outbreak, we aimed to determine their spatial connectivity to wintering areas in the CAF, densities of poultry, and reported outbreaks of HPAI H5N1 in this region. 

## 2. Results

### 2.1. Capture, Marking, and Sampling

We captured 28 ruddy shelducks and marked 26 with satellite platform transmitter terminals (PTTs: Table 1). We obtained 11,045 Argos locations derived from transmitter frequency doppler-shifts and 50,098 locations from global positioning system fixes (Table 1). Average transmitter lifespan was 13 months, but six transmitters were active >2 years and 1 worked >3 years. For the 26 marked shelducks, mean culmen length was 43.8 ± 2.3 mm (range 38.9–47.9), mean diagonal tarsus was 59.8 ± 4.6 mm (range 51.4–69.5), mean flat wing cord was 359 ± 17 mm (range 338–396), and their average mass was 1,331 ± 202 g (range 970–1725). Cloacal swabs, pharyngeal swabs, and blood samples were collected for all but two shelducks (1 unmarked adult female, and PTT #74817), but some samples were compromised from field to laboratory, resulting in tests on only 11 shelducks. All but one of those shelducks were negative, but that individual (shelduck #74822) had sandwich ELISA values above a 0.23 cutoff of 0.235 (oropharyngeal) and 0.249 (cloacal), indicating a positive result for influenza A. 

**Table 1:** Performance of satellite transmitters from 26 ruddy shelducks marked in 2007–2008 at Qinghai Lake, China through 15 May 2010. Total number of GPS and Argos locations is reported.

PTT	Sex	Age	Mass (g)	Capture Date	Last Transmission	Working Days	GPS Locations	Argos Locations	Total Locations
73042	F	A	1,135	9/12/2007	1/15/2008	126	314	84	398
74808	F	J	1,130	9/13/2007	3/21/2009	556	3274	762	4036
74809	F	J	970	9/14/2007	2/18/2008	158	800	148	948
74810	M	A	1,390	9/13/2007	Active	978	2946	913	3859
74812	M	J	1,510	9/13/2007	2/1/2008	142	137	16	153
74813	M	J	1,350	9/14/2007	12/10/2008	454	2207	412	2619
74816	M	A	1,600	9/16/2007	12/22/2008	464	1125	317	1442
74817	M	A	1,640	3/26/2007	1/3/2008	284	1299	157	1456
74818	M	A	1,270	9/13/2007	2/15/2008	156	772	92	864
74820	F	A	1,185	9/13/2007	4/21/2008	222	1675	308	1983
74822	F	A	1,070	9/14/2007	2/10/2009	516	1003	167	1170
74823	F	J	1,010	9/14/2007	11/16/2007	64	159	37	196
82095	F	A	1,295	3/28/2008	Active	782	3618	477	4095
82097	F	U	1,515	4/3/2008	6/6/2009	430	2919	756	3675
82099	M	A	1,580	4/3/2008	Active	776	4529	1048	5577
82100	M	A	1,470	4/3/2008	Active	776	2932	656	3588
82116	F	J	1,275	9/11/2008	1/14/2009	126	970	226	1196
82117	A	F	1,300	9/11/2008	Active	616	2530	518	3048
82118	M	A	1,495	9/15/2008	11/3/2008	50	264	43	307
82119	M	A	1,505	9/14/2008	7/12/2009	302	748	172	920
82120	M	J	1,160	9/13/2008	11/1/2008	50	361	80	441
82121	F	A	1,270	9/13/2008	6/9/2009	270	2351	506	2857
82122	M	A	1,725	9/13/2008	Active	612	3352	932	4284
82123	M	J	1,395	9/13/2008	11/21/2008	70	461	64	525
82126	M	J	1,155	9/12/2008	Active	614	4876	1244	6120
82127	F	A	1,210	9/13/2008	4/16/2010	581	4476	910	5386

### 2.2. Migratory Movements

#### 2.2.1. Major Use Areas in the Central Asian Flyway

**Figure 1:**
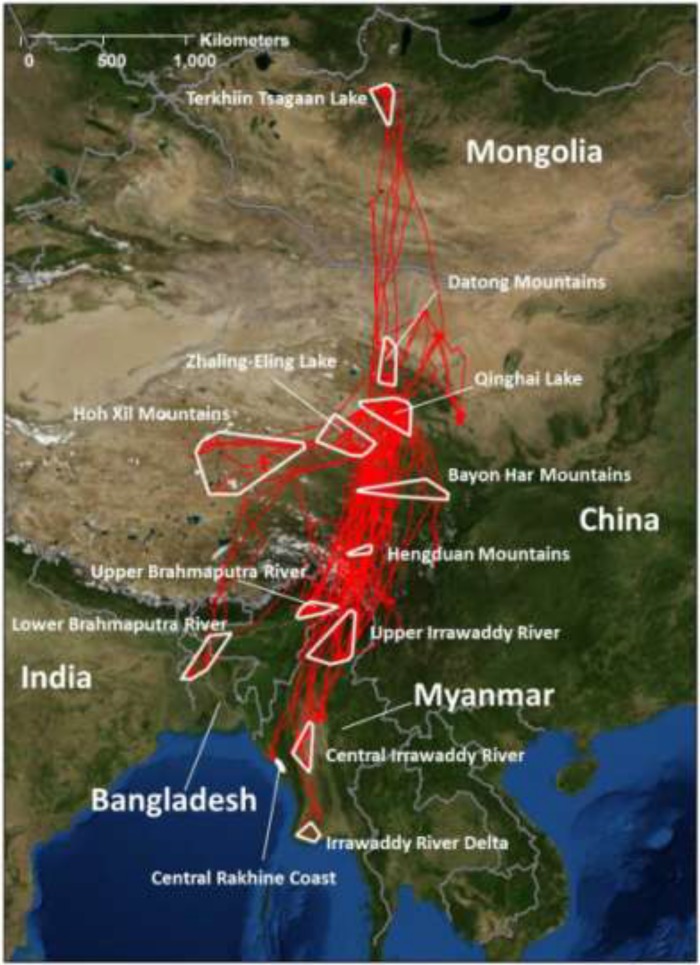
General movement patterns for 26 ruddy shelducks marked with satellite transmitters at Qinghai Lake, China. Study area and movement paths (red lines) for 26 ruddy shelducks marked with satellite transmitters in 2007–2008 and tracked through 15 May 2010. White polygons represent Minimum Convex Polygons (MCP) encompassing locations at breeding, wintering and stop-over areas.

**Figure 2:**
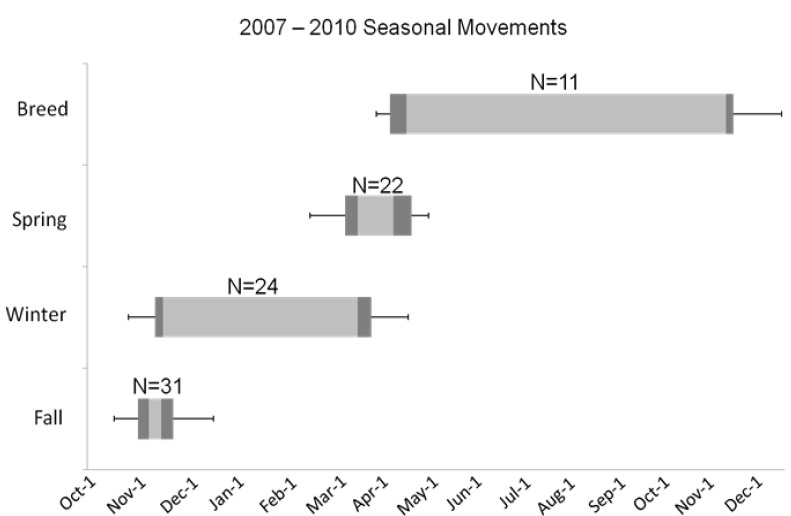
Seasonal movements (fall migration, winter, spring migration, and breeding/post-breeding) over three years for ruddy shelducks marked at Qinghai Lake, China. Seasonal movements were calculated for 20 shelducks with at least one full fall migration; birds with multiple annual cycles were treated as unique individuals in subsequent years. Light gray bars give median period start dates to median end dates, dark gray depicts the innermost 75th percentile, and error bars indicate first and last recorded dates within a period.

We mapped migration routes and stopover areas as well as breeding and wintering ground locations of 26 marked shelducks (Figure 1). A total of 31 fall migration movements and 22 spring migration movements were undertaken by 20 birds (Figure 2). The apparent breeding range of shelducks marked at Qinghai Lake was inferred by locations obtained during the reproductive period and described a large region extending from Tibet Autonomous Region (hereafter, Tibet) to central Mongolia (Table 2). During the fall and the spring, most shelducks used a 500-km wide migration corridor across central Qinghai Province. Their wintering grounds were distributed from Bangladesh, Myanmar, southern China, and northeast India (Figure 1). 

#### 2.2.2. Migration Chronology and Movement Rates

The breeding season for shelducks extended from April through October, while their fall migration began in mid-October and continued through mid-December (Figure 2). They were found on their wintering grounds between November and March before returning on spring migration beginning in mid-March. Surprisingly, only one or two major stopover areas were documented used during 10 of 31 fall migration movements and 15 of 22 spring migration movements (Table 3). 

Total migration distance ranged from 724–2,948 km (mean = 1724 ± 153 km). Longest distances between stopovers ranged from 771–2,686 km (mean =1612 ± 105 km), and stopovers lasted from 0.8–8.8 d (mean = 3.1 ± 0.3 d). Average fall migration as shelducks departed breeding areas in early Oct (mean = 4 Oct) and arrived in wintering areas a week later (mean = 14 Oct) was 8.8 d but ranged from 2–31 d with few stopovers (mean = 0.4 ± 0.1), while spring migration typically began on 30 March with arrival on breeding grounds of 16 May and lasted 22.9 ± 3.8 d (range= 2–52 d) with more stopovers (mean = 0.9 ± 0.2). 

**Table 2:** Migration stopover sites and wintering and breeding grounds of ruddy shelducks marked with satellite transmitters at Qinghai Lake, Qinghai Province, China from 2007–2008. Date ranges indicate arrival to departure, and sample sizes are number of unique individuals found at a site during a given time period. Primary breeding grounds (B), spring (S) and fall (F) migration stopover, and wintering grounds (W).

Site Name	Distance and Direction from Qinghai Lake (km)	Coordinates (Degrees Latitude, Longitude)	Time Period	N	Date Range	Mean Length of Stay (range in days)	Total Locations
Terkhiin Tsagaan Lake Region, Arkhangai Province, Mongolia	1240 N	48.09 N, 99.92 E	B 08-09	2	4/22–9/18	115 (98–149)	1615
Hoh Xil Mountain Region, PR China	570 SW	34.67 N, 91.60 E	B 08-09	3	7/8–10/26	95 (71–107)	1952
Datong Mountains Region, PR China	220 N	38.85 N, 99.44 E	B 08-09	4	5/5–7/27	59 (37–79)	864
Qinghai Lake Region, PR China	0-140 W, S	36.72 N, 99.02 E	B 07-09	10	4/2–12/13	174 (77–255)	5360
			S 08-10	7	3/8–4/25	13 (2–48)	259
			F 07, 09	2	10/23–12/6	13 (8–18)	17
Bayon Har Mountains Region, PR China	420 S	33.10 N, 100.52 E	S 08-09	4	3/12–4/29	23 (12–42)	576
Zhaling-Eling Lake Region, PR China	300 SW	35.42 N, 97.12 E	B 07-09	3	7/3–10/26	45 (28–110)	1147
			S 08-10	3	3/18–5/2	16 (4–22)	49
Hengduan Mountains Region, PR China	750 SW	30.28 N, 97.97 E	F 07-08	3	11/3–12/4	9 (2–16)	132
Upper Brahmaputra River, India	1140 SW	27.50 N, 95.03 E	W 07-09	3	11/2–4/15	102 (71–164)	4086
Lower Brahmaputra River and Ganges River, Bangladesh/India Border	1630 SW	25.05 N, 89.51 E	W 08-09	1	11/13–3/14	100 (78–121)	2446
Upper Irrawaddy River Region, Myanmar	1220 SW	26.18 N, 96.81 E	W 07-09	5	10/27–3/20	100 (71–144)	6154
Central Irrawaddy River Region, Myanmar	1850 SW	20.73 N, 94.92 E	F 08	1	11/3–11/29	26	358
			W 08-09	2	11/15–3/9	84 (78–96)	2524
Irrawaddy River Delta, Myanmar	1990 SW	19.75 N, 93.62 E	W 08	1	12/21–3/8	77	829
Central Rakhine Coast, Myanmar	2320 SW	16.23 N, 95.18 E	W 07	1	12/21–3/9	78	828

**Table 3:** Migration chronology and movement rates of ruddy shelducks marked at Qinghai Lake, China in 2007–2008. Dates, duration, and number of stopovers are reported for spring and fall migration. Longest leg (longest flight between two consecutive stationary areas, *i.e.*, stopovers, breeding, or wintering sites) and corresponding flight time, and total displacement (Euclidean distance from northern-most to southern-most points) are also given.

ID	Capture Date	Fall Migration				Spring Migration				Longest Leg			Total
		Depart Breeding	Arrive Wintering	Duration (d)	Stop-overs (N)	Depart Wintering	Arrive Breeding	Duration (d)	Stop-overs (N)	Leg Distance (km)	Time (d)	Rate (km/d)	Total Displacement (km)
73042	9/12/2007	10/31/2007	11/2/2007	3	0	na	na	na	na	831.0	1.4	594	724
74808	9/13/2007	11/23/2007	11/24/2007	2	0	3/12/2008	5/2/2008	52	1	1,422.0	2.3	610	2,686
		11/14/2008	11/18/2008	5	0	3/20/2009	na	na	na	1,331.4	4.1	326	
74809	9/14/2007	11/24/2007	11/26/2007	3	0	na	na	na	na	1,068.9	1.8	583	1,095
74810	9/13/2007	11/9/2007	12/7/2007	29	1	3/12/2008	5/2/2008	52	1	2,431.0	2.8	859	3,461
		11/7/2008	11/16/2008	10	0	3/19/2009	4/22/2009	35	2	2,240.7	8.8	256	
		10/18/2009	11/14/2009	28	1	3/4/2010	4/28/2010	56	1	1,763.8	1.6	1,085	
74813	9/14/2007	11/1/2007	11/2/2007	2	0	4/7/2008	5/4/2008	28	1	1,047.2	2.2	483	1,474
		11/1/2008	11/2/2008	2	0	na	na	na	na	888.7	0.8	1185	
74816	9/16/2007	11/8/2007	11/20/2007	13	1	3/15/2008	4/9/2008	26	2	1,833.3	2.3	801	1,754
		11/13/2008	11/24/2008	12	1	na	na	na	na	770.9	2.7	289	
74817	3/26/2007	10/28/2007	11/1/2007	5	0	na	na	na	na	2,009.6	3.5	574	1,633
74818	9/13/2007	10/27/2007	10/30/2007	4	0	na	na	na	na	1,141.6	3.0	381	1,205
74820	9/13/2007	11/12/2007	11/19/2007	8	1	3/28/2008	na	na	na	1,881.7	2.3	808	2,372
74822	9/14/2007	11/26/2007	12/20/2007	25	1	3/17/2008	5/1/2008	46	1	979.3	6.5	151	1,144
82095	3/28/2008	11/15/2008	11/16/2008	2	0	4/8/2009	4/10/2009	3	0	1,071.8	1.0	1,072	1,362
		11/14/2009	11/19/2009	6	0	3/30/2010	4/14/2010	16	1	1,742.5	5.0	349	
82097	4/3/2008	11/14/2008	11/17/2008	4	0	3/22/2009	3/25/2009	4	0	2,481.5	3.1	802	2,178
82099	4/3/2008	11/29/2008	12/10/2008	12	1	3/10/2009	4/2/2009	24	1	1,612.8	2.5	645	1,616
		12/14/2009	12/16/2009	3	0	2/25/2010	2/28/2010	4	0	1,940.1	2.2	895	
82100	4/3/2008	11/6/2008	11/9/2008	4	0	3/25/2009	4/11/2009	18	1	2,947.5	7.0	419	2,271
		11/12/2009	11/15/2009	4	0	2/19/2010	3/28/2010	38	2	2,033.6	3.2	642	
82116	9/11/2008	11/3/2008	11/4/2008	2	0	na	na	na	na	1,288.5	1.5	859	1,347
82119	9/14/2008	11/14/2008	11/16/2008	3	0	4/8/2009	4/12/2009	5	0	2,008.8	4.6	441	1,233
82121	9/13/2008	11/1/2008	12/1/2008	31	1	3/24/2009	4/13/2009	21	1	2,050.1	3.9	523	2,655
82122	9/13/2008	11/1/2008	11/12/2008	12	2	3/23/2009	4/21/2009	30	2	1,759.3	2.4	746	1,546
		10/30/2009	11/2/2009	4	0	4/15/2010	4/17/2010	3	0	1,647.0	1.4	1,145	
82126	9/12/2008	11/6/2008	11/7/2008	2	0	4/24/2009	4/25/2009	2	0	1,112.6	1.3	890	1,415
		10/25/2009	10/27/2009	3	0	3/20/2010	4/6/2010	18	1	1,089.9	3.0	363	
82127	9/13/2008	11/14/2008	12/8/2008	25	1	4/6/2009	4/9/2009	4	0	2,620.3	4.1	637	2,381
		11/9/2009	11/13/2009	5	0	3/14/2010	4/1/2010	19	2	934.7	3.5	264	

#### 2.2.3. Space Use and Distribution

**Figure 3:**
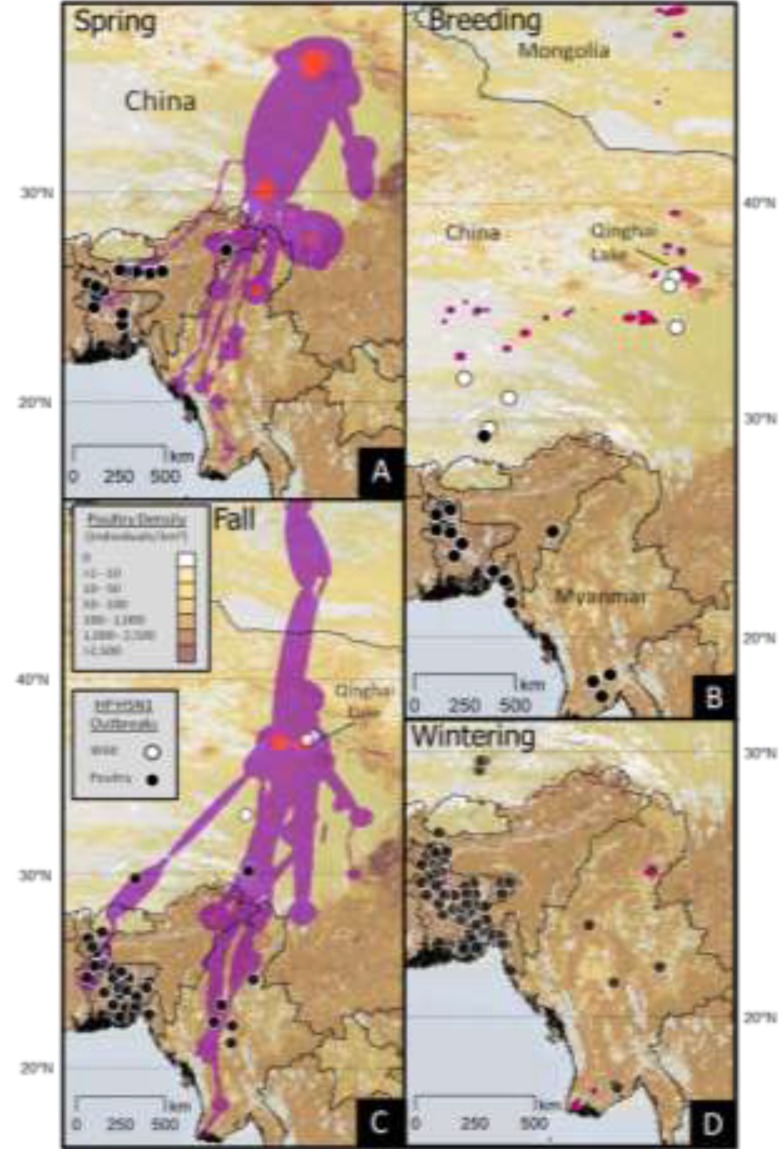
Utilization distributions for ruddy shelducks marked at Qinghai Lake, China, 2007–2008; shown in relation to poultry density (brown shading) and HP H5N1 outbreaks, occurring in wild birds (white circles) and poultry (black circles), from September 2003 to April 2010. Brownian bridge utilization distributions describe (**A**) spring (10 March–4 May) and (**C**) fall (27 October–20 December) migration movements. Fixed kernel home ranges depict population level (**B**) breeding (5 May–26 October) and (**D**) wintering (21 December–9 March) areas. Two shading levels (red, purple) for all utilization distributions indicate isopleths containing 95% and 99% of total locations, respectively.

**Figure 4:**
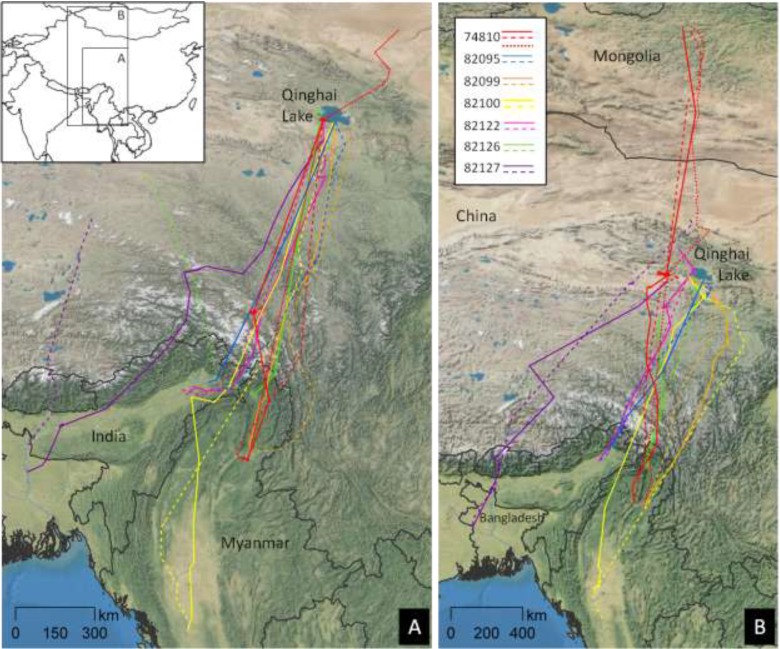
Routes for ruddy shelducks marked at Qinghai Lake, China and followed for at least two seasonal migratory cycles. Repeated migratory routes for seven ruddy shelducks marked at Qinghai Lake, 2007–2008. All seven shelducks completed two or more full migration movements (fall and spring). Paths are given for (**A**) fall (2007–2009) and (**B**) spring (2008–2010) migration movements, with solid, dashed, and dotted lines as first, second, and third journeys, respectively.

Breeding locations extended across a wide area from Qinghai Lake north to Mongolia and southwest toward Tibet (Figure 3). During the fall, Brownian bridge utilization distribution (BBUD) models described a central migration corridor extending to Myanmar with a smaller southeast branch toward Sichuan and a southwest branch toward Bangladesh (Figure 3). We found little movement and very small home ranges for shelducks during the winter (Figure 3). Individuals, wintering in Bangladesh and Myanmar, followed separate spring migration routes (Figure 3) until they reached the primary spring migration corridor in southern Tibet. Seven individuals completed multiple migration cycles (Figure 4), and they showed strong site fidelity to their wintering grounds, migration corridors, and breeding grounds. Shelduck 74810 (Figure 4) was paired to sheduck 74808 (not shown) and they migrated together to north-central Mongolia where they likely bred.

### 2.3. HPAI H5N1 Outbreaks

**Figure 5:**
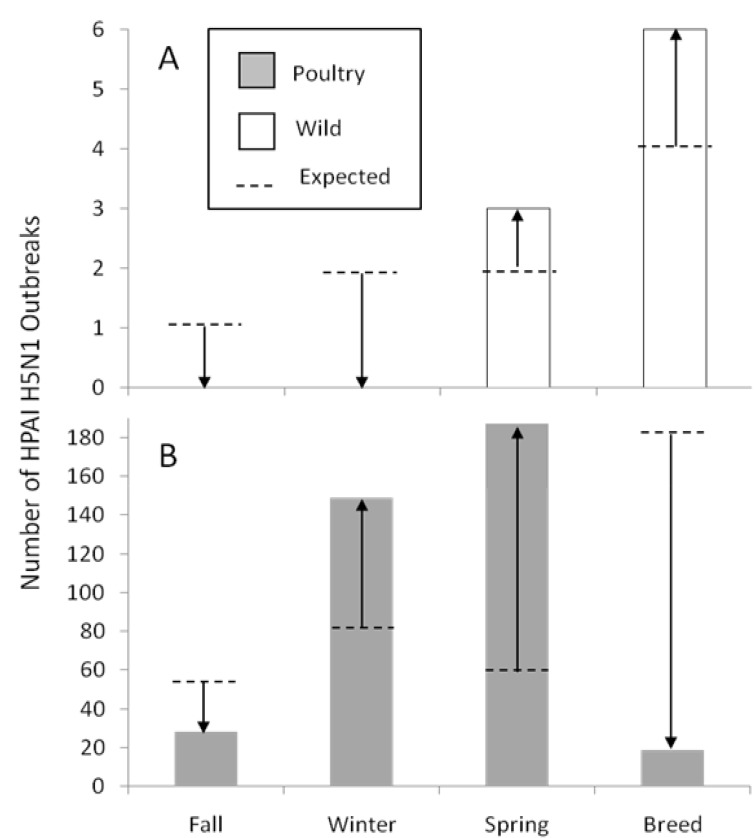
Comparison of wild and domestic outbreaks from 2003–2010 by seasonal period in the life cycle of ruddy shelducks. Observed *vs.* expected values differed for both (**A**) wild bird and (**B**) domestic poultry outbreaks when tested separately (wild: *P* = 0.043, Fisher Exact Test; poultry: *P* < 0.0001, Chi-Squared Test). Expected numbers were calculated under the assumption that outbreaks are proportional to the number of days within the fixed seasonal period (fall migration = 55 d; winter = 79 d; spring migration = 56 d; and breeding = 175 d).

From 2003–2010, 392 outbreaks (nine in wild birds, 383 in poultry) were reported within the CAF. Most poultry outbreaks occurred in the winter and the spring and were clustered in and around Bangladesh, with others in Myanmar, northeast India, and southern China (Figure 3). Wild bird outbreaks were distributed across the Qinghai-Tibetan Plateau including four outbreaks clustered at Qinghai Lake. When we compared the seasonal rate of outbreaks to the length of the migration, breeding and wintering periods (Figure 5), we found that wild bird outbreaks occurred during spring migration (3) and breeding periods (6), while more poultry outbreaks occurred during spring migration (187) and wintering (149) rather than the fall (28) and breeding season (19).

#### 2.3.1. Relation to Ecological Parameters

Forward and backward stepwise AIC*c* analyses (Table 4, Table 5) both indicated the same top model for domestic poultry outbreaks which included wetland, cropland, latitude, developed lands, and poultry density as important predictors (combined AIC*c* weight = 45.6%). Although the distribution of shelducks was included in one of the top models for domestic poultry (Table 4), it was not one of the most important parameters (Table 5). Results were similar for wild bird outbreaks and identified best‑fitting models as those including latitude and developed lands (combined AIC*c* weight = 49.6%).

**Table 4:** Ranking of candidate corrected Akaike Information Criterion (AIC*c*) models for domestic and wild outbreaks in the Central Asian Flyway. Sample size, variables, −2 log-likelihood, AIC*c*, delta AIC*c*, and Akaike weights are reported. Ranking of AIC*c* models to compare domestic poultry and wild bird highly pathogenic avian influenza H5N1 disease outbreak sites against random locations within the migratory pathway of ruddy shelducks marked at Qinghai Lake, China, 2007–2008. Backward and forward stepwise methods were used to select the best model for each wild and domestic outbreaks with different combinations of the seven predictor variables, presented with the full model (all variables) and the null model (intercept only). Variables include percent wetland (Wetl), cropland (Crop), developed land (Dev), and grassland (Grass), as well as latitude (Lat), poultry density (Pdens), and a seasonal utilization distribution for satellite-marked ruddy shelducks (RUSHUD).

Outbreak Type	Model	N	K	−2 Log- likelihood	AIC*c*	ΔAIC*c*	Akaike Weight (%)
Domestic	Wetl, Crop, Lat, Dev, Pdens	4213	6	1,757.8	1,769.8	0	45.6
	Wetl, Crop, Lat, Dev, Pdens, Grass, RUSHUD	4213	8	1,757.1	1,773.1	3.3	8.9
	Null Model (intercept only)	4213	0	2,566.9	2,566.9	797.0	0.0
Wild	Lat, Dev	99	3	33.5	39.5	0	49.6
	Wetl, Crop, Lat, Dev, Pdens, Grass, RUSHUD	99	8	30.2	46.2	8.1	0.9
	Null Model (intercept only)	99	0	60.3	60.3	20.6	0.0

**Table 5:** Best-fit models and significant parameters for poultry and wild bird outbreaks in the Central Asian Flyway. Best-fit models with parameter values for poultry and wild bird HPAI H5N1 disease outbreaks within the Central Asian Flyway and migratory pathway of ruddy shelducks marked at Qinghai Lake, China, 2007–2008. Models were selected by forward and backward stepwise selection using AIC*c* values for entry and removal from models. Variables include percent wetland (Wetl), cropland (Crop), developed land (Dev), and grassland (Grass), as well as latitude (Lat), poultry density (Pdens), and a seasonal ruddy shelduck utilization distribution (RUSHUD) for satellite marked shelducks.

Model	Parameter	Β	SE	Wald χ^2^	Pr > χ^2^
Poultry Outbreaks	Intercept	−0.2415	0.6307	0.1466	0.7018
	Poultry Density	0.0001	0.0001	2.7242	0.0988
	Wetland	2.1	0.5557	14.2805	0.0002
	Cropland	3.1258	0.1953	256.1308	<0.0001
	Latitude	−0.1637	0.0261	39.4072	<0.0001
	Urban	15.7376	1.3640	133.1154	<0.0001
Wild Outbreaks	Intercept	−25.2017	7.3469	11.7665	0.0006
	Latitude	0.7044	0.2158	10.6519	0.0011
	Urban	21.0892	13.0902	2.5955	0.1072

#### 2.3.2. Correlation and Regression Tree

**Figure 6:**
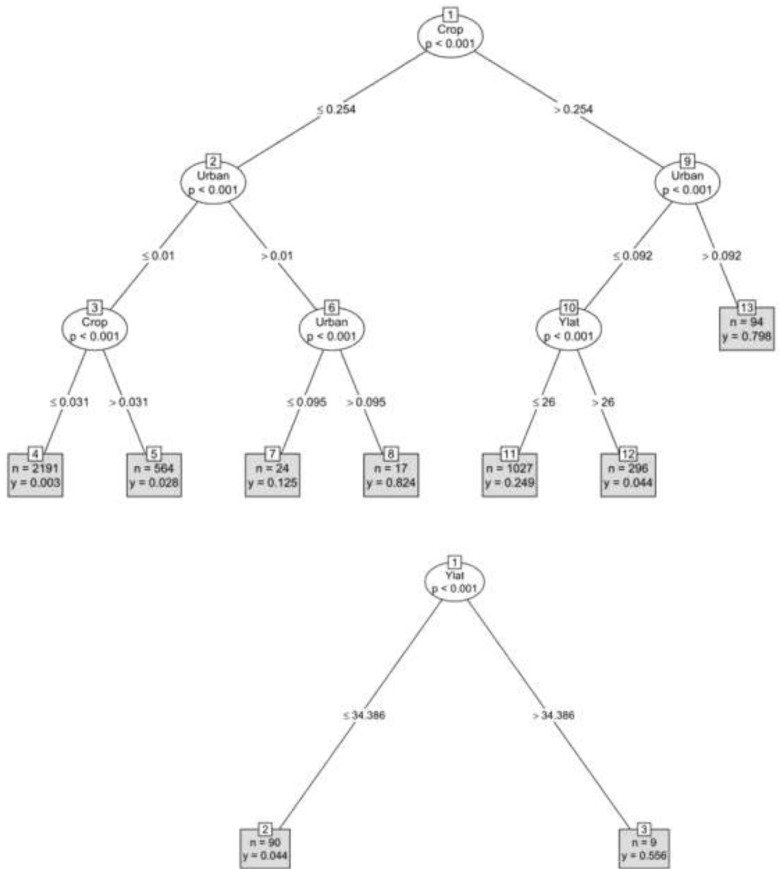
Correlation and regression tree for wild bird and domestic poultry outbreaks in the Central Asian Flyway. Expected numbers were calculated assuming outbreaks were proportional to seasonal period length. Values in circles indicate the variable and if differences were significant, while numbers along the tree branches indicate separation points for that variable in order of highest importance from top-to-bottom.

CART (correlation and regression tree) results for 383 poultry outbreaks indicated that most occurred when cropland percent exceeded 25.4%, urban areas were <9.2%, and the latitude exceeded 26 degrees (Figure 6). The nine wild bird outbreaks were found at latitudes exceeding >34.3 degrees, but none of the other ecological parameters was useful in determining where they would occur.

## 3. Experimental Section

### 3.1. Study Area

We conducted fieldwork to capture shelducks at the Qinghai Lake National Nature Reserve, Qinghai Province, in north-central China (36.82° N, 99.81° E). Qinghai Lake is a large, high-altitude saltwater lake (4,500 km^2^, 3,200 m above sea level) on the eastern edge of the Qinghai-Tibet Plateau. The lake is frozen from November through March. The climate is arid and cool, with a short rainy season from June-August (0.35 m annual average rainfall) from the East Asian monsoon, with most rainfall occurring from May to September [18]. The lake is a closed basin fed by 25 freshwater streams, the majority of which have intermittent flow [19]. The 495,000 ha Qinghai Lake Reserve was established in 1975, and in 1992 listed as a Wetland of International Importance [20]. Qinghai Lake is an important migration area for waterbirds in the CAF, and is recognized as a key breeding area for many waterbird species, including shelducks [21,22].

### 3.2. Capture, Virology Sampling, and Marking

We captured shelducks in late March–early April of 2007 and 2008, and in September of 2007 and 2008 using monofilament noose sets and a remotely-activated net launcher (Coda Enterprises, Mesa, AZ, USA). Upon capture, birds were placed in individual cloth bags and promptly processed. Capture, handling, and marking procedures were approved by the U.S. Geological Survey’s (USGS) Patuxent Wildlife Research Center Animal Care and Use Committee (ACUC) and University of Maryland Baltimore County Institutional ACUC (Protocol EE070200710). We collected standard morphometrics (mass, flat wing chord, culmen, and diagonal tarsus [23]; and recorded age and sex. All but two individuals were sampled for avian influenza (cloacal and tracheal swabs, blood serology) following standard procedures [24]. Virology analyses included: (1) type A influenza with a sandwich ELISA test (Optical Density 630 above 0.23 as positive), (2) H5 subtype with RT-PCR [25], and (3) H5, H7, H9, and H10 antibodies with hemagglutinin inhibition [26]. Virology samples were processed by the Chinese Academy of Sciences, Wuhan Institute of Virology (for further details, see [27]). 

We targeted adults and equal numbers of males and females for marking. Shelducks were marked with 22 or 30-g, solar-powered GPS platform terminal transmitters (PTT; Microwave Telemetry, Columbia, Maryland USA). PTTs were attached dorsally with a double-threaded backpack harness made of Teflon ribbon (Bally Ribbon Mills, Bally, PA, USA). Transmitter packages averaged <2.4% of the bird’s body mass. Each bird was released immediately after marking near its capture location. PTTs were programmed to transmit for 10-h periods every 2 days and to take GPS locations every 2 h. Location data were uploaded to satellites every 2 d (CLS America Inc., Maryland, USA). Locations also were calculated by the Doppler-effect shift in transmission frequency during satellite overpasses. Each Doppler-derived location included a location quality index of >1,000 m (class 0), 350–1,000 m (1), 150–350 m (2), and ≤150 m (3). We used a filtering algorithm (Douglas Filter Version 7.03 [28,29]), to identify and remove implausible locations based on distance moved, movement rate, and turning angle. We used ArcGIS 9.3 (Environmental Systems Research Institute, Inc., Redlands, CA, USA) and Google Earth 5.0 (Google, Mountain View, CA, USA) to plot and analyze telemetry locations.

### 3.3. Telemetry and Movements

We plotted the movements of marked shelducks from capture until last transmission (or 15 May 2010). We analyzed seasonal movement patterns assigning each location to breeding (including post‑breeding molt), wintering, and spring and fall migration. Fall migration was defined as the last location on the breeding grounds prior to long distance longitudinal movements until the first location on wintering grounds when longitudinal movements became localized. Spring migration was defined as the converse, and the intervening periods were considered breeding and wintering seasons. We defined migratory stopovers as areas used during spring or fall migration in which a shelduck moved ≤20 km in a 24 h period. We calculated the cumulative distance and time it took for an individual to complete each migration leg (the path between two consecutive stopover locations) for all shelducks that completed at least one fall migration. We identified the longest leg (km) for each shelduck as an estimate of greatest distance travelled between stationary periods. The total migration distance for each individual was defined by the Euclidian distance between its northernmost and southernmost location. For shelducks with multiple years of telemetry data, we plotted migration routes and stopovers to examine path overlap and site fidelity. We used ArcGIS 9.3 and Hawth’sTools [30] to perform these analyses. We reported movement statistics as the mean ± SE unless indicated otherwise.

### 3.4. H5N1 Outbreaks and Risk Factors

HPAI H5N1 outbreak data for 2003 through April 2010 were obtained from the United Nations Food Agriculture Organization’s (UNFAO) Emergency Prevention System for Transboundary Animal and Plant Pests and Diseases (EMPRES-i) database which includes all official Office International des Epizooties (OIE) outbreak reports, supplemented by the People’s Republic of China Ministry of Agriculture Prevention and Control of Avian Influenza database [31] as in Prosser *et al.* [32]. Outbreak events were cross-checked between the two databases and outbreak locations were imported into a GIS framework for analysis. We included only those outbreaks that occurred within a minimum convex polygon encompassing all recorded shelduck telemetry locations, and considered only domestic animal and wild bird outbreaks (excluding human cases).

We analyzed outbreaks in conjunction with poultry density, land cover, and probability distributions of shelduck presence. Poultry density data with 1 km resolution were obtained from the UNFAO via the Geonetwork [33]. Methodology and sources of the estimates are described in the FAO’s Gridded Livestock of the World [34] and in Takekawa *et al.* [35]. For land cover features, we used the Moderate-resolution Imaging Spectroradiometer (MODIS)/Terra Land Cover Classification [36]. We used the 500 m resolution MCD12Q1 layer with the International Geosphere-Biosphere Programme classification scheme (IGBP Type 1), which is divided into 17 classes. We condensed the IGBP Type 1 scheme into 6 broad land cover categories: (1) water, (2) grassland, (3) cropland, (4) wetland, (5) developed, and (6) other, including natural vegetation types as well as barren or sparsely vegetated areas.

### 3.5. Statistical Analyses

To relate shelduck migration movements to potential risk factors, we created Brownian Bridge Utilization Distribution models (BBUD; [37]) on the basis of telemetry locations and compared these with locations of H5N1 outbreaks. The BBUD model assumes locations are temporally dependent and uses length of time between locations, along with measurable location error, to weight spatial probability of occurrence. These features prove most useful for migration when animals are moving large distances in short periods of time [37]. We created BBUD models for fall and spring migration from the first migration movement for each individual with Animal Space Use [38]. Population-level migration routes for spring and fall were calculated as the mean probability of occurrence across individuals [37]. We averaged across individuals to define seasonal periods and calculate population‑level migration corridors for the spring and fall; thus population-level spring and fall migration seasons were defined using the earliest departure and latest arrival dates of marked birds. For Brownian bridge and home range analyses, population-level fixed seasons were defined as: breeding (5 May–26 October; 175 days); fall migration (27 October–20 December; 55 days); wintering (21 December–9 March; 79 days); and spring migration (10 March–4 May; 56 days). 

Fixed-kernel home range utilization distributions were created for the sedentary seasons (breeding and winter) to describe shelduck space use. We applied the likelihood cross-validation (CVh) method to obtain kernel smoothing parameters [39], and home ranges were developed for breeding and wintering with Animal Space Use [38]. Shelducks were weighted equally by including the same number of randomly selected locations for each individual. Probabilistic utilization distribution models for ruddy shelducks (RUSHUD) were created for each of the four seasons using Brownian bridge movement models for spring and fall migration and fixed kernel home range models for breeding and non-breeding seasons.

We also examined temporal aspects of outbreaks for wild birds and domestic poultry by comparing the observed and expected number of outbreaks based on the length of the season for each outbreak type. Expected numbers were calculated under the assumption that outbreaks were proportional to the number of days within a seasonal period. We used a Fisher’s exact test for these comparisons to accommodate small sample size [40,41].

### 3.6. Outbreak Characteristics

We used forward and backward stepwise selection with corrected Akaike’s Information Criteria (AIC*c*) values as criteria for entry into and removal to examine the relationship of shelduck movements, habitat risk factors, and poultry or wild bird outbreaks. We compared outbreak and non-outbreak (random) locations in logistic regression models [42,43]. In forward selection, we avoided collinearity by restricting a variable from entering the model if its absolute correlation coefficient with any variable already in the model was >0.8. In backward selection, we retained the variable from each pair that produced the lower AIC*c* when the other variable was excluded. The remaining variables had <0.8 absolute correlation. We calculated odds ratios (OR) for variables in the best models to determine if the relationship was more (OR > 1) or less likely (OR < 1).

We applied a second-order analysis of AIC*c* = −2(log-likelihood) + 2*K*(*N*/*N* − *K* − 1), where *K* is the number of fitted parameters including variance and *N* is the sample size [44,45]. We considered the model with the smallest AIC*c* to be the most parsimonious [44,45]. We used the AIC*c* differences between the best model and the other candidate models (ΔAIC*ci* = AIC*ci* − minimum AIC*c*) to determine the relative ranking of each model. Akaike weights (*wi* = exp [−ΔAIC*ci*/2]/Σexp [−ΔAIC*ci*/2]) were calculated to assess the evidence that the selected model was the best Kullback-Leibler model [44,45].

Covariates included latitude (Lat), shelduck utilization distribution (RUSHUD), poultry density (Pens), cropland (Crop), wetland (Wetl), and developed land (Urban) for both poultry and wild bird models. Ten random locations were defined for each outbreak to provide a comparison [42]. Random locations were drawn proportionally from the spatial extent of outbreaks in each season. Data were extracted from 5 km buffers around outbreak and random points using ArcGIS 9.3 Spatial Analyst (Environmental Systems Research Institute Inc., Redlands, CA, USA) and Hawth’s Tools zonal summary; AIC*c* stepwise logistic regressions were performed with SAS GLM (family = binomial, link = −logit; SAS Institute 2004, Cary, NC, USA).

We used separate correlation and regression tree (CART) analyses [46,47,48] to determine binomial breakpoints when parameters were useful in predicting poultry and wild bird outbreaks. Parameters included land cover percentages for wetland, cropland, developed land, and grassland; latitude; poultry density; and seasonal ruddy shelduck utilization distributions. For each outbreak location, we generated 10 pseudorandom locations within the flyway where outbreaks had not been reported [42], and we determined the value for parameters when outbreaks and random points were separated.

## 4. Discussion and Conclusions

Si *et al.* [49] have suggested that global H5N1 outbreaks correspond with bird migration patterns at the scale of the flyway. Our earlier high-resolution studies of ducks in the East Asian Flyway (EAF) did show correlation of outbreak locations with migration routes of ducks from Poyang Lake in southeast China, but a closer analysis of their movements showed that they were not temporally correlated with the timing of the outbreaks [35]. In this study of the CAF, our Akaike Information Criterion analysis indicated that outbreaks were correlated with land cover, latitude, and poultry density. This result is not surprising since poultry are abundant in the southern wintering areas while densities are very low or absent in much of the northern reaches of the CAF, although a few wild bird outbreaks did occur in northern areas (Figure 3; and see Suppl. Material in [7]). Although Qinghai Lake is located at the intersection of the CAF and EAF, shelduck movement remained entirely within the CAF during fall migration. Shelduck movements were included in the top two models, but they were not a top parameter selected in AIC*c* stepwise regression results. Consistent with our earlier studies on other waterfowl species in China [4,32,35], and contrary to early hypotheses of H5N1 movement across flyways by wild birds [12], there is no evidence that shelducks moved from EAF areas in southeastern China where HPAI H5N1 is thought to have originated [50] to the CAF.

Outbreaks have persisted in the CAF (see summaries in: [8,32,35]), including at the original wild bird outbreak area of Qinghai Lake. In comparison to bar-headed geese [32], another species of H5N1 concern, shelducks had a longer migration (1,724 km *vs.* 1,373 km) and longer jumps between stopovers (1,612 km *vs.* 708 km) of shorter duration (2.1 d *vs.* 3.1 d). Their fall migration was later (departure 9 Nov *vs.* 14 Oct) but shorter (8.8 *vs.* 26.2 d), while their spring migration was earlier (departure 14 Mar *vs.* 30 Mar) but longer (22.9 d *vs.* 5.0 d). With mean movement rates of 280 km/d and maximum rates of 1,200 km/d, shelducks had the potential to travel large distances before succumbing to disease. Kwon *et al.* [51] detected asymptomatic periods of five to nine days for ruddy shelduck challenged with A/chicken/Korea/IS/06 (H5N1) HPAI. Thus compared with bar-headed geese, shelducks had a higher potential to transmit the virus farther from the wintering areas in the spring. However, they used very few stopover areas during the spring migration, suggesting that after departing wintering areas, they had little chance of intermixing with concentrations of wild birds or domestic poultry in the CAF.

Most wintering shelducks marked at Qinghai Lake were found wintering in the eastern Indian subcontinent to the Bay of Bengal in areas where domestic and wild bird flocks intermix and vaccination is largely absent [52,53]. On the wintering grounds, shelducks were found feeding on riverbanks [13] and in farmlands during the midwinter [54] where they could intermix with domestic bird flocks. In contrast, bar-headed geese wintered in northern India or Lhasa (southern Tibet) in areas with higher biosecurity and vaccination (although bar-headed goose farms occur near Lhasa [32]).

Our analyses relied on accurate outbreak reporting rates, but outbreaks may be under-reported, especially for wild birds or for remote northern migration and breeding areas with few human observers. In addition, our sample of marked shelducks ideally would represent the greater shelduck population, however, the expense of satellite telemetry restricts marking to a relatively small sample of individuals, and a general lack of knowledge of subpopulations for this species precluded stratified sampling. Different individuals within a species may exhibit different migration strategies [55], and this may result in wide variation in exposure and presence of avian influenza viruses [55]. Thus, identification of the particular subpopulation or group that migrates in an area where HPAI H5N1 persists [56] is essential to better understand the dynamics of the repeat outbreak occurrences.

Examining outbreaks in the CAF separately for domestic and wild birds revealed differences in pattern with poultry outbreaks beginning in the winter and extending through the breeding season, with wild bird outbreaks occurring only during the spring and breeding periods [7,25], this study. Poultry outbreaks that typically precede wild bird outbreaks in the CAF may suggest spillover from poultry to wild birds [8]. Some of the marked shelducks migrated long distances and flew more rapidly. Those individuals that follow a migration strategy to “jump” between areas [57] may have a higher potential of spreading AIVs from wintering to breeding areas. Other species of wild birds may be involved in spread; although after extensive sampling, waterfowl species are still considered the reservoir for low pathogenic avian influenza [3]. Thus, the leading hypothesis to explain persistent circulation at Qinghai Lake [21,58], where there are no poultry, remains spillover from poultry in wintering areas carried by wild birds during northward migration in the CAF.

The migration results presented here were in general agreement with analyses that used finer biweekly-utilization-distributions [59] and indicated spatial and temporal agreement with outbreaks [8]. In that analysis, spatial and temporal overlap with outbreaks was closer for shelducks (46.2%) than bar-headed geese (19.2%) [8], although phylogenetic analyses with bar-headed geese suggested correlation of their movements to distribution of AIV subtypes [32]. In this analysis, we found that BBUDs for shelducks were in AIC*c* models associated with outbreak conditions, but BBUDs by themselves were not significant parameters. These differences suggest that focusing on small temporal changes in distribution may be critical in making the link between outbreaks and the movements of wild birds when sample sizes are adequate. Applying broad movement corridors in models of AIV spread [49] may not be sufficient to properly correlate outbreaks with the temporally changing movements of migratory birds.

It is quite likely that there is a time lag in the arrival of wild birds with the observation of outbreaks. The time it typically may take for introduction from wild birds to poultry once exposed is not known. Modeling of potential AIV spread by a widespread waterfowl, the common teal (*Anas crecca*), indicated that persistence in the wild bird population would only occur with excretion durations of longer than seven days or survival in water [60]. This compares with laboratory studies [51,61] that found shelducks were highly susceptible to HPAI H5N1 with 100% mortality and had a shorter period of shedding of only 5.5 days. 

Host traits may influence the occurrence and role of wild birds of avian influenza transmission [62]. Bar-headed geese are a social species that mate for life and nest in colonies, but shelducks mate annually and nest as pairs [63]. Cross-species infections have not been well-studied, but the opportunity seems evident in the case of these two species that often intermingle at stopover and breeding areas along Qinghai Lake [64]. 

Shelducks may not represent the wide range of species that may be involved in the spread of this disease. Although shelducks were killed during the 2005 Qinghai Lake outbreak, other abundant species also died including the Pallas’ gulls (*Larus ichthyaetus*) which has a similar migration pattern [65] and also migrates long distances. Species such as the northern pintail (*Anas acuta*) were found in staging areas for long periods following HPAI H5N1 outbreaks in Japan before moving rapidly northward on their spring migration [66].

In summary, limited availability of ecological data to better understand emerging diseases such as HPAI H5N1 has been pointed out as a serious deficiency that limits our ability to understand and model disease ecology [67,68]. Our ecological studies are an attempt to improve the availability of such information for better understanding the spread of viruses.
